# Supplementary material for: Integrated Single-cell Analysis Uncovers Regulatory Logic of Cranial Ectoderm Development
Source: bioRxiv. 2025 Dec 17:2025.12.17.694990. Preprint. [Version 1] doi: 10.64898/2025.12.17.694990 (PMC12724563; doi:10.64898/2025.12.17.694990)
Supplement: 11 [file NIHPP2025.12.17.694990v1-supplement-11.pdf]

bioRxiv preprint doi: https://doi.org/10.64898/2025.12.17.694990; this version posted December 17, 2025. The copyright holder for this preprint (which was not certified by peer review) is the author/funder. This article is a US Government work. It is not subject to copyright under 17 USC 105 and is also made available for use under a CC0 license.

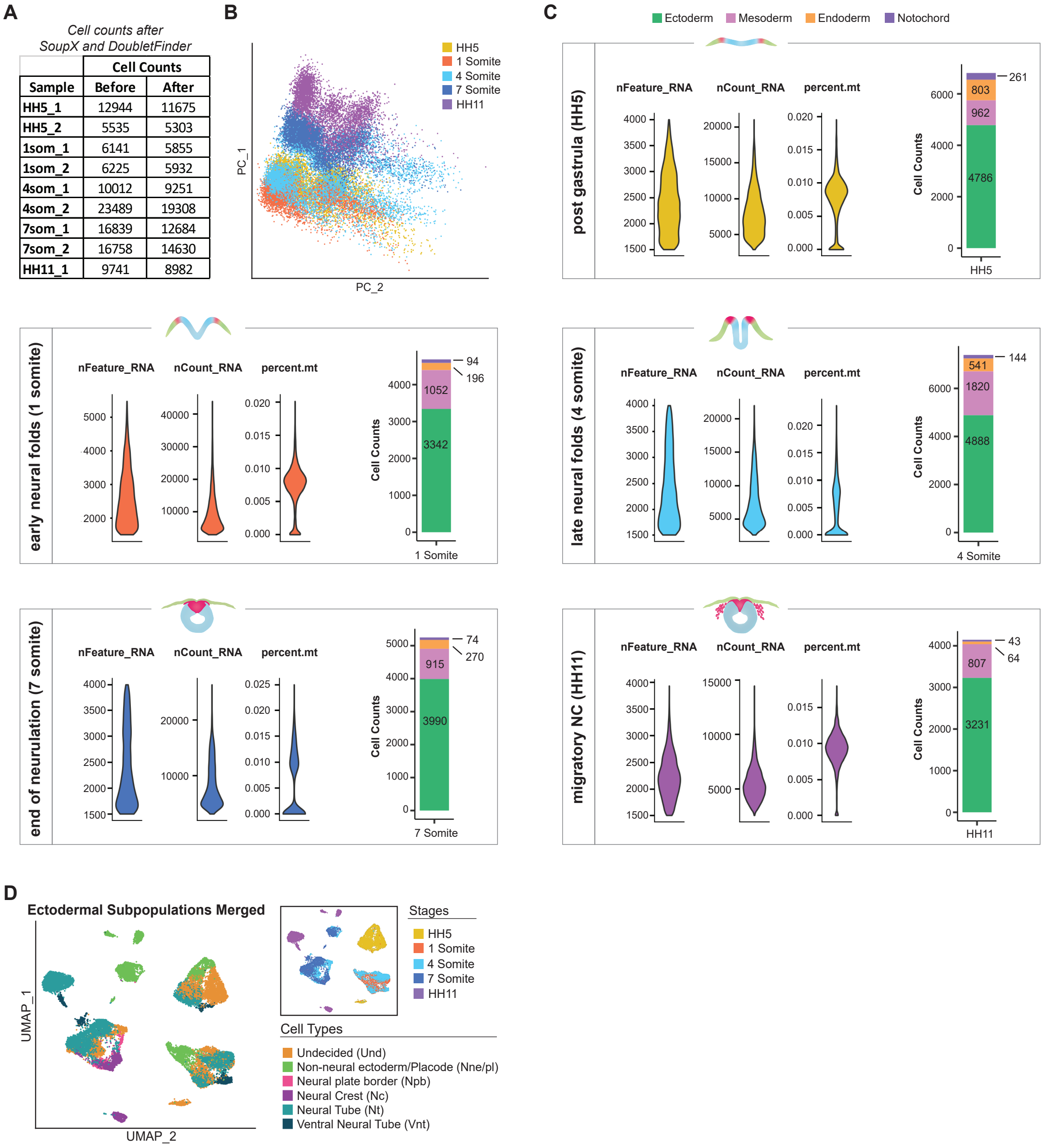

	Cell Counts	
**Sample**	**Before**	**After**
**HH5_1**	12944	11675
**HH5_2**	5535	5303
**1som_1**	6141	5855
**1som_2**	6225	5932
**4som_1**	10012	9251
**4som_2**	23489	19308
**7som_1**	16839	12684
**7som_2**	16758	14630
**HH11_1**	9741	8982

Supplemental Figure 1

A) Cell counts of the scRNA-seq samples pre- and post-cleanup using SoupX for ambient RNA removal and DoubletFinder for doublet identification. **B)**, PCA analysis of merged samples, demonstrating no apparent batch effects across the dataset. **C)**, Violin plots for each of the five developmental stages, illustrating nFeature_RNA, nCount_RNA, mitochondrial content, and cell

bioRxiv preprint doi: https://doi.org/10.64898/2025.12.17.694990; this version posted December 17, 2025. The copyright holder for this preprint (which was not certified by peer review) is the author/funder. This article is a US Government work. It is not subject to copyright under 17 USC 105 and is also made available for use under a CC0 license.

distribution across germ layers. **D)** Merged ectodermal subsets from all five stages, color-coded by cell type and stages separately.

bioRxiv preprint doi: https://doi.org/10.64898/2025.12.17.694990; this version posted December 17, 2025. The copyright holder for this preprint (which was not certified by peer review) is the author/funder. This article is a US Government work. It is not subject to copyright under 17 USC 105 and is also made available for use under a CC0 license.

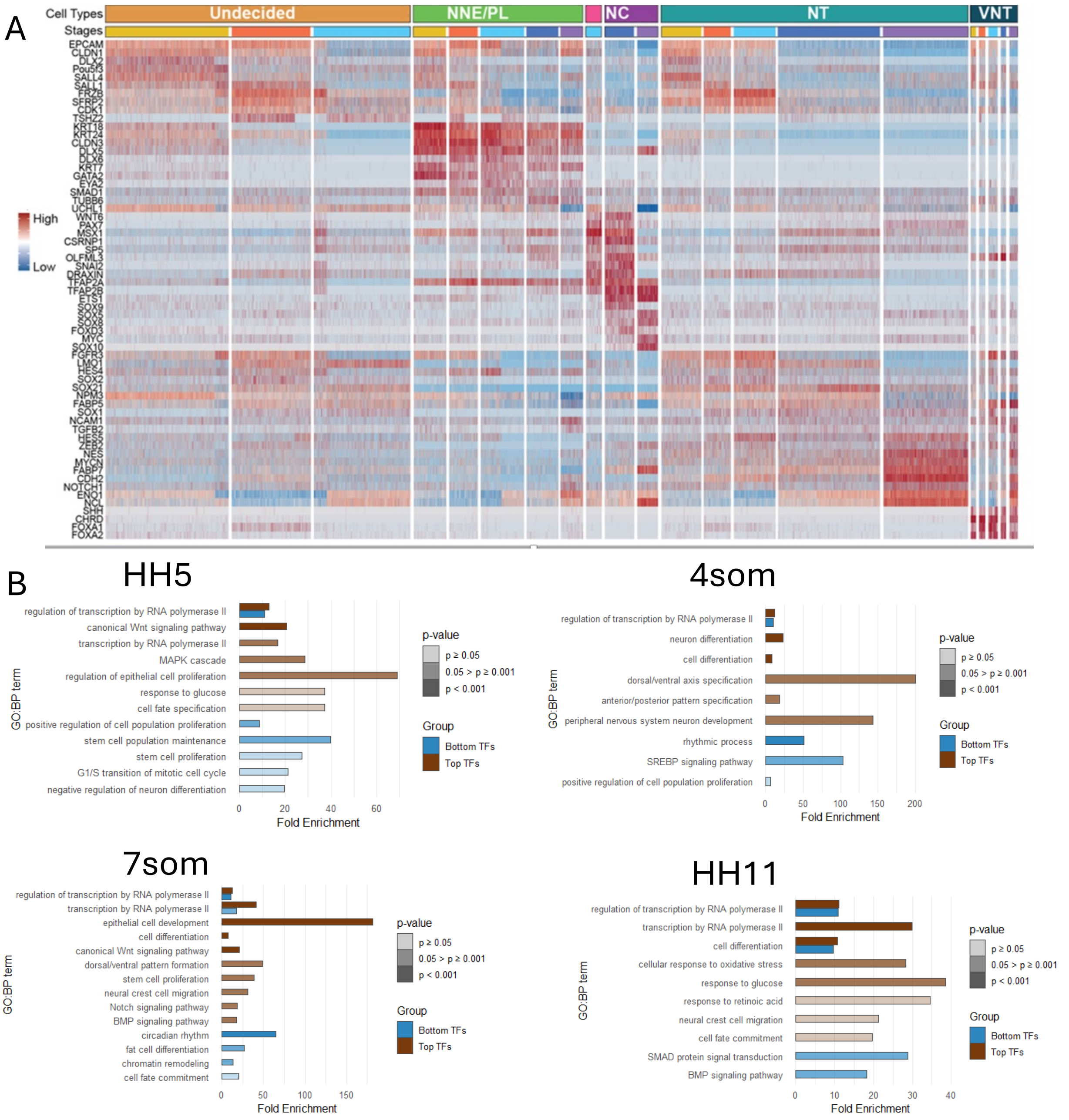

Supplemental Fig 2

A) Heatmap showing top differentially expressed genes per 21 unique idents. Full list of genes is in supplemental Table 2 (minimum percent = 0.25, log fold-change threshold = 0.2).

Top gene ontology terms consisting of genes with top and bottom (n= 30 each) gene activity scores at **B)** HH5, **C)** 4somite **D)** 7 somite **E)** HH11. Bars show fold enrichment, with significance represented as –log10(Benjamini FDR).

bioRxiv preprint doi: https://doi.org/10.64898/2025.12.17.694990; this version posted December 17, 2025. The copyright holder for this preprint (which was not certified by peer review) is the author/funder. This article is a US Government work. It is not subject to copyright under 17 USC 105 and is also made available for use under a CC0 license.

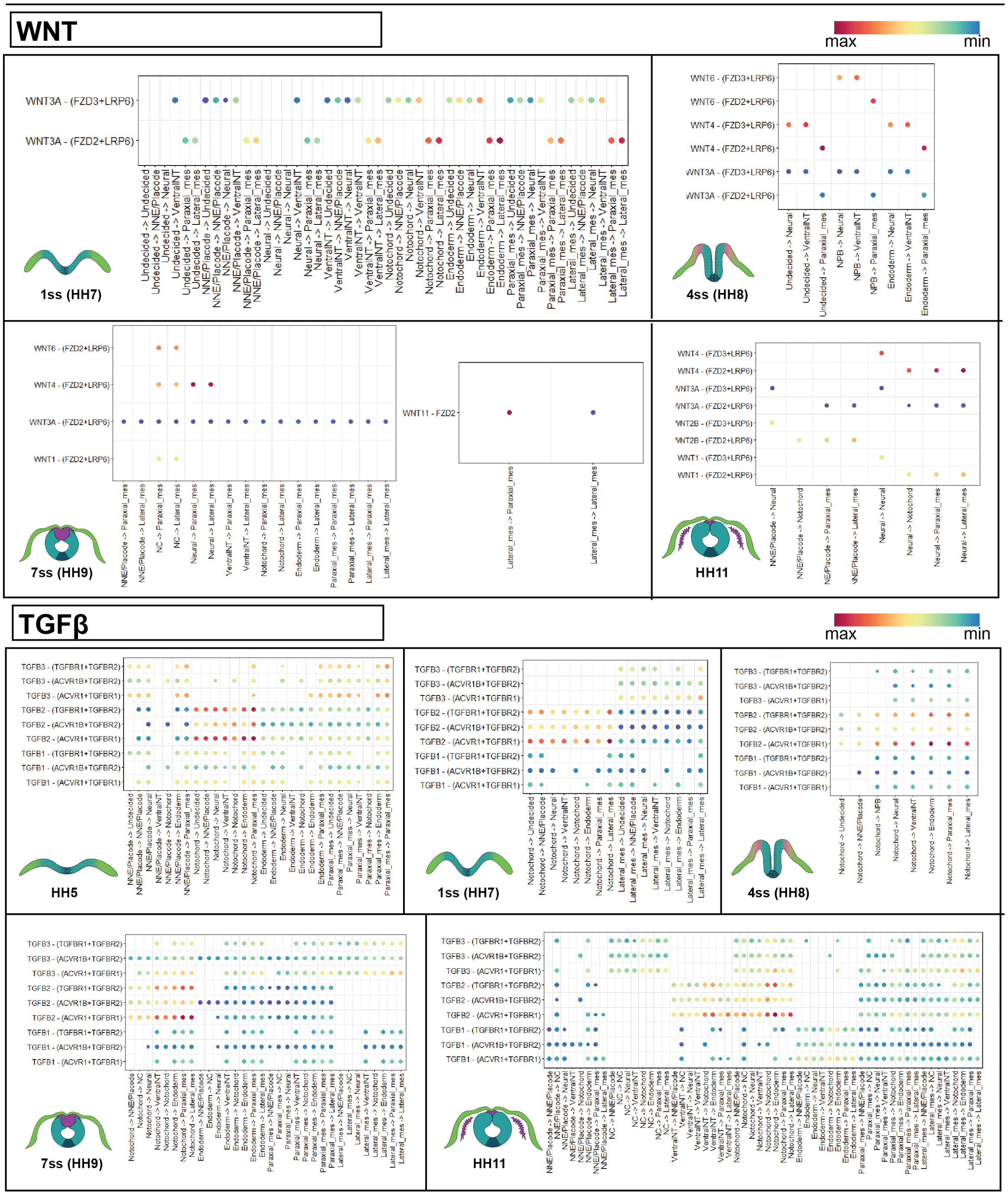

Supplemental Fig 3
Individual Cell Chat ligand-receptor pair interaction predictions for Wnt (1som-HH11) and TgfB-signaling (HH5-HH11).

bioRxiv preprint doi: https://doi.org/10.64898/2025.12.17.694990; this version posted December 17, 2025. The copyright holder for this preprint (which was not certified by peer review) is the author/funder. This article is a US Government work. It is not subject to copyright under 17 USC 105 and is also made available for use under a CC0 license.

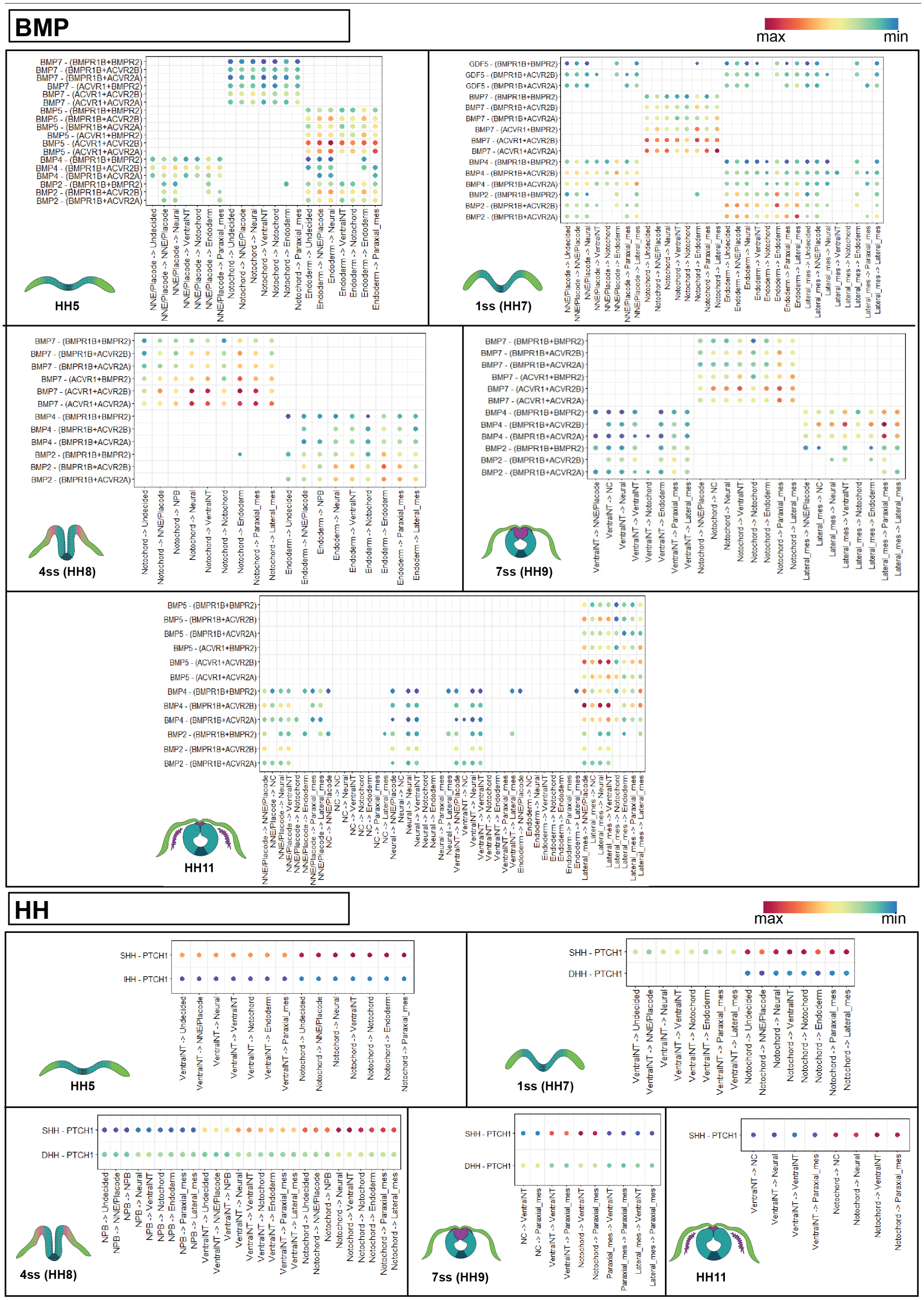

bioRxiv preprint doi: https://doi.org/10.64898/2025.12.17.694990; this version posted December 17, 2025. The copyright holder for this preprint (which was not certified by peer review) is the author/funder. This article is a US Government work. It is not subject to copyright under 17 USC 105 and is also made available for use under a CC0 license.

Supplemental Fig 4
Individual Cell Chat ligand-receptor pair interaction predictions for BMP and Hedgehog-signaling pathways (HH5-HH11).

bioRxiv preprint doi: https://doi.org/10.64898/2025.12.17.694990; this version posted December 17, 2025. The copyright holder for this preprint (which was not certified by peer review) is the author/funder. This article is a US Government work. It is not subject to copyright under 17 USC 105 and is also made available for use under a CC0 license.

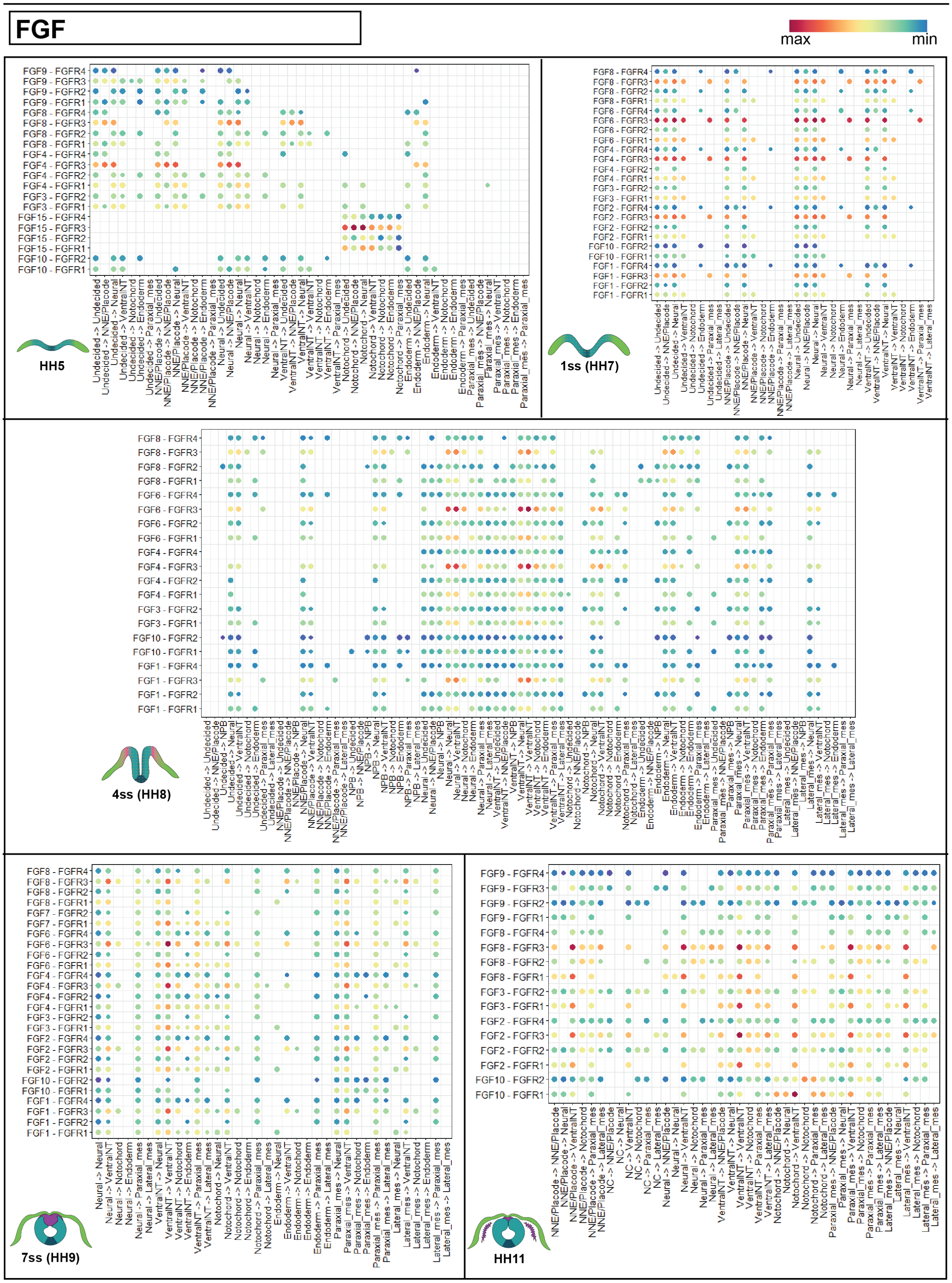

bioRxiv preprint doi: https://doi.org/10.64898/2025.12.17.694990; this version posted December 17, 2025. The copyright holder for this preprint (which was not certified by peer review) is the author/funder. This article is a US Government work. It is not subject to copyright under 17 USC 105 and is also made available for use under a CC0 license.

Supplemental Fig 5
Individual Cell Chat ligand-receptor pair interaction predictions for the FGF signaling pathway (HH5-HH11).

bioRxiv preprint doi: https://doi.org/10.64898/2025.12.17.694990; this version posted December 17, 2025. The copyright holder for this preprint (which was not certified by peer review) is the author/funder. This article is a US Government work. It is not subject to copyright under 17 USC 105 and is also made available for use under a CC0 license.

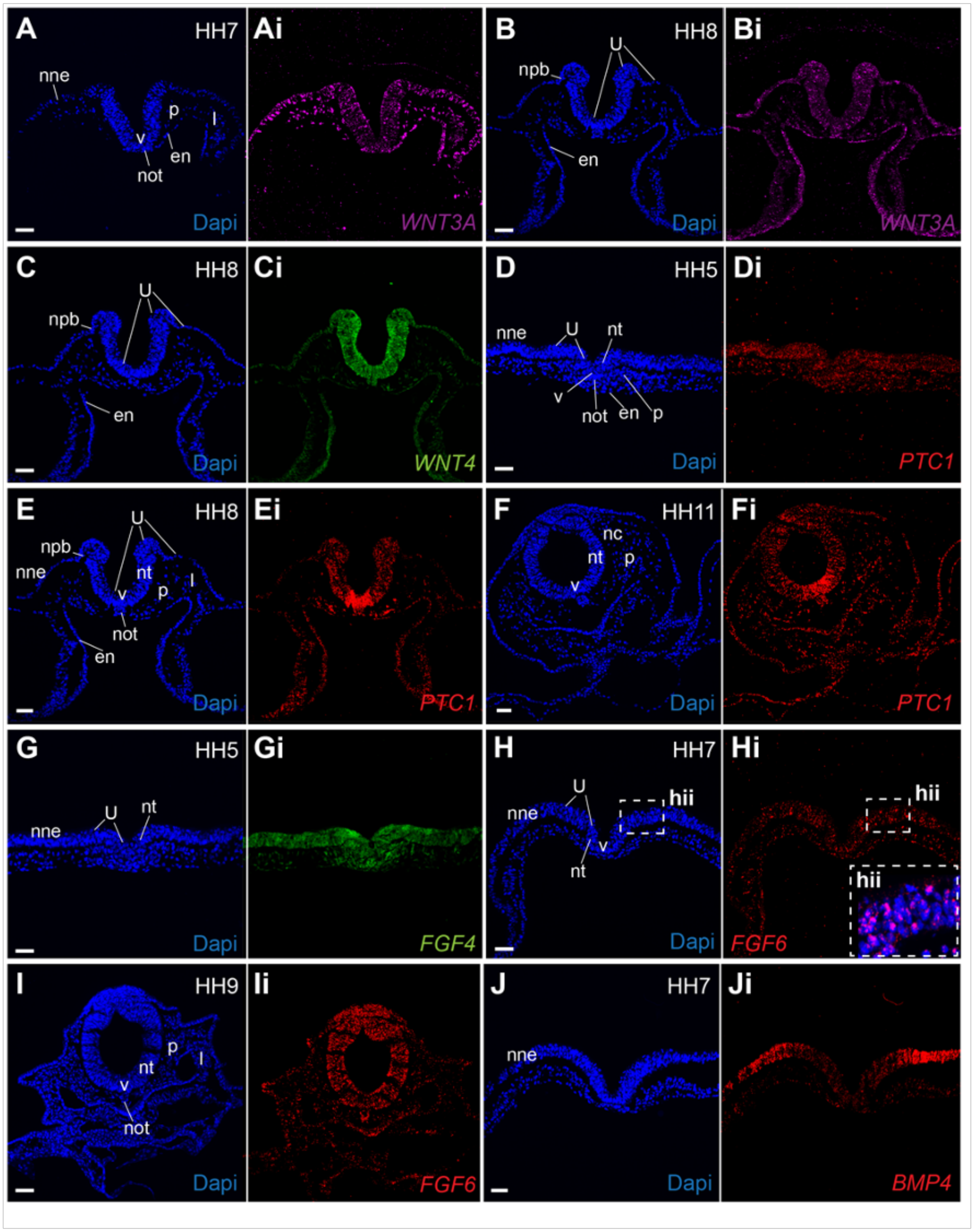

Supplemental Figure 6

HCR-amplified fluorescent *in situ* hybridization-based validation of Cell Chat predictions not found in the literature. **A)** *Wnt3A* at HH7 **B)** *Wnt3A* at HH8 **C)** *Wnt4* at HH8, **D)** *Ptc1* at HH5, **E)** *Ptc1* at HH8, **F)** *Ptc1* at HH11 **G)** *Fgf4* at HH5, **H)** *Fgf6* at HH7 **I)** *Fgf6* at HH9 **J)** *Bmp4* at HH7. Scale Bar 50µm.

bioRxiv preprint doi: https://doi.org/10.64898/2025.12.17.694990; this version posted December 17, 2025. The copyright holder for this preprint (which was not certified by peer review) is the author/funder. This article is a US Government work. It is not subject to copyright under 17 USC 105 and is also made available for use under a CC0 license.

Gene	Locations	References
**FZD3**	Could not be validated in the literature at these stages.	
**LRP6**	Could not be validated in the literature at these stages.	
**WNT4**	HH8–HH11; neural tube	Hollyday, 1995
**WNT3A**	HH6; neural plate and primitive streak	http://geisha.arizona.edu/geisha/search.jsp?entrez_gene=448977
**WNT11**	HH10 - lateral mesoderm	Cauthen 2001
**WNT6**	HH8–HH9; neural tube, non-neural ectoderm	Schubert 2002, http://geisha.arizona.edu/geisha/search.jsp?entrez_gene=461439
**FZD2**	HH7–9: uniformly expressed.<br>HH8–9: neural folds, somites, non-neural ectoderm.<br>HH10/11: neural tube, notochord, paraxial mesoderm, lateral mesoderm, somites	http://geisha.arizona.edu/geisha/search.jsp?entrez_gene=454452, Stark 2000, Cauthen 2001
**BMP5**	HH5–HH10: endoderm<br>HH8–HH10/11: lateral plate mesoderm	Yamagishi et al 2001.
**ACVR2B**	HH5–HH11: broadly expressed	Stern 1995, McCabe 2007, http://geisha.arizona.edu/geisha/search.jsp?entrez_gene=456530
**ACVR1B**	Could not be validated in the literature at these stages.	
**ACVR1**	HH5–HH11: uniformly expressed.	Cooly 2014
**AVCR2A**	HH5: mesoderm, notochord<br>HH7: lateral plate mesoderm, endoderm, neural plate<br>HH8: neural folds, non-neural ectoderm, endoderm<br>HH11: notochord, paraxial mesoderm, lateral plate mesoderm, non-neural ectoderm, uniform low level expression	Stern et al 1995, McCabe 2007, http://geisha.arizona.edu/geisha/search.jsp?entrez_gene=461860
**BMP2**	HH5-HH8: endoderm	Somi 2004, Andree 1998
**BMP4**	HH7: non-neural ectoderm<br>HH8-HH10: lateral mesoderm	Streit 1998, Somi 2004, http://geisha.arizona.edu/geisha/search.jsp?entrez_gene=459056
**BMP7**	HH9: notochord	http://geisha.arizona.edu/geisha/search.jsp?entrez_gene=449323
**BMPR2**	HH7: neural plate, endoderm	http://geisha.arizona.edu/geisha/search.jsp?entrez_gene=455159
**BMPR1B**	HH11: somites, neural tube, notochord, paraxial mesoderm	http://geisha.arizona.edu/geisha/search.jsp?entrez_gene=363845
**FGF1**	Could not be validated in the literature at these stages.	
**FGF15/19**	HH5: notochord	Kurose et al 2004
**FGF2**	HH5-11: neural tube, non-neural ectoderm<br>HH11: paraxial mesoderm, notochord	Karabagli 2002
**FGF3**	HH11: Paraxial mesoderm, neural tube, non-neural ectoderm	Karabagli 2002
**FGF4**	HH5: neural<br>HH7: uniformly expressed, neural plate<br>HH8: neural<br>HH9/10: notochord, neural tube, endoderm and paraxial mesoderm	Paxton et al 2010, Bothe 2011, http://geisha.arizona.edu/geisha/search.jsp?entrez_gene=456309
**FGF6**	reported as not expressed at these stages	Kumar 2012
**FGF7**	Could not be validated in the literature at these stages.	
**FGF8**	HH4: primitive streak and early mesoderm<br>HH7-11: endoderm, neural folds, placode<br>HH11: paraxial mesoderm	Karabagli 2002, http://geisha.arizona.edu/geisha/search.jsp?entrez_gene=461905
**FGFR3**	HH5-HH11: neural plate/tube<br>HH5: lateral epiblast<br>HH7: neuroepithelium and paraxial mesoderm	Lunn 2007, Nishita 2011
**FGFR1**	HH5-11: neural tube	Lunn 2007, Nishita 2011
**FGF10**	HH11: notochord, prechordal plate	Karabagli 2002, Bothe 2011
**TFGB2**	HH5-8: neural tube, notochord	Cooly 2014
**TGFB3**	HH5-6: weakly throughout the epiblast and mesoderm	Cooly 2014
**TGFBR1**	HH5-11: broadly expressed at low levels. Endoderm, lateral and paraxial mesoderm ectoderm, notochord and neural tube	Cooly 2014
**TGFBR2**	HH7-11: paraxial mesoderm, lateral mesoderm	Cooly 2014
**SHH**	HH5-8: ventral neural tube, notochord	Aglyamova 2007, Pearse 2001, Brito 2006, http://geisha.arizona.edu/geisha/search.jsp?entrez_gene=449446
**PTCH1**	HH5: ectoderm, mesoderm<br>HH8-11: ventral NT, lateral plate mesoderm<br>HH11: notochord	Aglyamova 2007, Pearse 2001, http://geisha.arizona.edu/geisha/search.jsp?entrez_gene=450001

Supplemental Fig 7

Literature sources for published *in situ* hybridization data that validates Cell Chat predictions.
